# Bridging Infant Mental Health Screening and Intervention from NICU to Home: A Single-Center Quality Improvement Project

**DOI:** 10.3390/bs16101940

**Published:** 2026-10-19

**Authors:** Melissa C. Carson, Tamara Matic, Rita V. Burke, Christine B. Mirzaian, Jennifer Yuen, Douglas Vanderbilt, Marian E. Williams

**Affiliations:** 1 Division of General Pediatrics, Children’s Hospital Los Angeles, Los Angeles, CA 90027, USA; cmirzaian@chla.usc.edu (C.B.M.); mwilliams@chla.usc.edu (M.E.W.); 2 Department of Pediatrics, Keck School of Medicine of University of Southern California, Los Angeles, CA 90033, USA; rita.burke@med.usc.edu (R.V.B.); dvanderb@stanford.edu (D.V.); 3 Department of Psychology, Children’s Hospital Los Angeles, Los Angeles, CA 90027, USA; tmatic@chla.usc.edu (T.M.); jyuen@chla.usc.edu (J.Y.); 4 Department of Population and Public Health Sciences, Keck School of Medicine of University of Southern California, Los Angeles, CA 90032, USA; 5 Division of Developmental-Behavioral Pediatrics, Children’s Hospital Los Angeles, Los Angeles, CA 90027, USA

**Keywords:** infant mental health, universal, dyadic, NICU, high-risk infant follow-up clinic, early intervention

## Abstract

Having an infant admitted to a NICU increases parental anxiety, depression and traumatic stress, heightens infants’ developmental and mental health risk, and may disrupt infant–parent relationships. Early Connections is a dyadic, trauma-informed universal infant mental health program implemented at Children’s Hospital Los Angeles. Child–Parent Psychotherapy-informed dyadic psychological intervention is offered during admission in the NICU. The Parent Navigator model utilizes a parent with lived NICU experience to support linkage to early intervention as families transition home. Finally, a psychologist provides infant mental health screening and tiered intervention in the interdisciplinary outpatient Newborn Follow-up Program (NFP), a high-risk infant follow-up clinic. This manuscript describes preliminary findings from a single-center quality improvement project to explore implementation of the program from December 2025 through March 2026. Of the 63 (55.3%) families who met with the NICU psychologist, 88.9% had at least one infant mental health family session and 65.1% had more than one infant mental health session during their child’s admission. Of the 66 (57.9%) supported by the parent navigator, 90.9% completed applications for early intervention for their infant; future follow-up will determine the outcome of the application for services. Based on outpatient infant mental health screening conducted with 85 patients (50.5% of those who had appointments), 23.6% of infants and 40.0% of toddlers screened in the NFP were at risk for social–emotional concerns, suggesting there may be a continued need for ongoing screening and intervention following discharge from intensive care. As the needs of families and infants change across inpatient and outpatient medical settings, it is important to offer multiple opportunities to screen needs flexibly and provide tiered intervention.

## 1. Introduction

The neonatal intensive care unit (NICU) is an inherently stressful medical environment that creates barriers and disruptions to parent–infant attachment and bonding. Parents of NICU infants commonly experience acute stress, grief and loss, and postpartum mental health disorders, including postpartum anxiety, depression, and posttraumatic stress (Hansen et al., 2025; Malouf et al., 2024; Osborne et al., 2025; Trumello et al., 2018). PTSD symptoms related to NICU experiences have been found to occur in up to one-third of parents whose infant was in the NICU (McKeown et al., 2023); such symptoms serve as a mediator to parenting stress (Suttora et al., 2014) and can impact the developing parent–infant relationship. There is also a growing body of literature that suggests that the psychological wellbeing of both families and healthcare providers is essential to optimal outcomes for the infant (de Visser et al., 2023; Hall et al., 2015; Paraiso-Pueyo et al., 2026; Yui et al., 2026).

Infant mental health is the emerging ability of a young child to form close, secure relationships; manage a range of feelings; and explore and learn within family, cultural, and community systems (Zero to Three Infant Mental Health Task Force Steering Committee, 2001). In the NICU, holding an infant mental health perspective amid medical complexity requires focusing on infant regulation in the context of nurturing relationships and reflecting on the infant’s and family’s experiences to support infant development and a successful transition home from the hospital (Browne, 2021). To foster nurturing relationships, it is essential to support the parent–infant dyad during the NICU hospitalization and beyond. When considering best practice guidelines and the impact of parental psychological distress on the parent–infant relationship and parental role attainment, there is often a need for dyadic intervention in addition to addressing parental mental health concerns (Givrad et al., 2021). However, in a scoping review of dyadic interventions for infants hospitalized in the NICU, few studies included dyadic psychotherapy interventions (Aokalani et al., 2026).

As embedded psychology positions have grown in NICUs, there has been increased awareness about the importance of supporting parent–infant relationships during hospitalization. However, there are also opportunities to support the dyadic relationship in outpatient follow-up appointments (Alderdice & Redshaw, 2015), such as in high-risk infant follow-up programs. In a review of nurturing factors to promote infant mental health in the NICU and interventions to foster NICU parent–infant relationships, authors summarized that interventions should begin as soon as at-risk families are identified and continue after hospital discharge (Givrad et al., 2021). Outpatient medical appointments, which can be numerous for NICU graduates, provide additional opportunities to support dyadic relationships and infant mental health through screening and intervention.

Rates of depression for mothers whose infants were admitted to the NICU were found to be 38% within the first year of discharge from the hospital, and higher depressive symptoms were associated with a lower level of attachment with their infant (Leahy-Warren et al., 2020). Also, mothers of preterm infants have been found to have a higher level of postpartum depression symptoms for a longer period following the birth of their child, indicated by ratings taken when the child was approximately 5–12 months (Leahy-Warren et al., 2020). This demonstrates the ongoing risk for families and the need for infant mental health screening that extends beyond the initial hospitalization to outpatient settings where NICU graduates receive medical care.

Infants who have lengthy NICU hospitalizations or painful and intrusive procedures are also at risk of experiencing medical traumatic stress. Medical trauma has been defined as, “Exposure to a single, or series of medical event/s related to injury/illness, painful or invasive medical intervention and/or other hospital experiences that may be perceived by the child and/or caregiver as threatening, overwhelming or frightening” (De Young et al., 2021, p. 740). Relatedly, pediatric medical traumatic stress is a non-diagnostic framework describing the reaction of children and their parents to illness, medical procedures, and treatments, including physical and psychological symptoms such as re-experiencing, avoidance, and hyper-arousal (Kazak, 2006). Parents can often buffer their infants from stress; however, when an infant must be separated from their parents during hospitalization, they are at increased risk. While an infant’s stress response may be expected during the crisis in the NICU, if the stress response continues after discharge from the hospital it may lead to problems with regulation and sleep and challenge parental confidence or the parent–infant relationship. A triad of cumulative early life NICU experiences (stress, parental separation, and pain) may influence an infant’s risk for adverse behavioral, cognitive, and emotional outcomes, thus providing further evidence of the need for screening NICU graduates in outpatient settings (D’Agata et al., 2016). Additionally, approximately 30% of young children, ages birth through six, have been found to develop medical traumatic stress symptoms within the first month of experiencing an illness or injury, with up to 10% developing posttraumatic stress disorder (De Young et al., 2021). Thus, repeated opportunities for social–emotional screening are important for infants and toddlers who have complex medical needs and ongoing medical procedures extending well beyond their NICU admission. Infants who are born preterm or experience other medical complications necessitating NICU admission are at high risk for developmental delays and/or developmental disabilities (Allen, 2008; Behrman et al., 2007; Pappas et al., 2015), which can further contribute to parenting stress (Treyvaud et al., 2011). Early intervention services can help improve developmental outcomes of NICU graduates (McManus et al., 2012; Orton et al., 2024); however, connection to this community-based resource after NICU discharge can be challenging for families (Little et al., 2015).

Given the increased risk to the infant, parent, and the parent–infant relationship in families coping with a NICU admission and complex medical follow-up, a dyadic approach that can be applied flexibly across inpatient and outpatient medical settings is essential. The Stein Tikun Olam Early Connections Program is a dyadic, trauma-informed, universal infant mental health program implemented at Children’s Hospital Los Angeles (CHLA). This program builds on work described previously to develop a continuum of infant mental health care from the prenatal period to hospitalization to follow-up (Driver et al., 2021; Lakatos et al., 2024). New developments in the program have focused on seeking to provide universal infant mental health services to all parent–infant dyads while hospitalized in the NICU, rather than the prior model of a referral-based program. In addition, an infant mental health screening and intervention protocol was added to the Newborn Follow-up Program (NFP), a medical high-risk infant follow-up clinic, with the aim of offering the service to all families in the clinic. The program’s new screening and intervention approach will be described, including the addition of the role of a parent navigator who supports families as they are discharged from the NICU to outpatient follow-up services, focusing on early intervention. Infant mental health interventions have been adapted or developed aligning with universal, targeted, or clinical levels (Kazak, 2006) to provide support tailored to a family’s level of current need. The aim of the current manuscript is to examine time-limited data from a new, single-center screening and intervention project to support families of infants as they transition from the NICU to outpatient settings.

## 2. Materials and Methods

### 2.1. Quality Improvement Design

This manuscript is based on a prospective single-center quality improvement project designed to evaluate program implementation, identify factors supporting successful implementation, and determine areas for improvement. The Plan–Do–Study–Act (PDSA) framework was used to guide the iterative testing and refinement of changes within the local clinical setting (Langley et al., 2009). Specifically, the framework guides our process of introducing the screening approach, evaluating completion rates and implementation barriers, and refining workflow based on what was learned.

### 2.2. Setting and Context

Children’s Hospital Los Angeles (CHLA) is an urban pediatric academic medical center. The patient population at CHLA NICU is diverse demographically, with more than 72% of Latinx ethnicity. Approximately 76% of families have Medicaid and 24% have private insurance.

The settings included in this project include a 58-bed, level IV NICU providing care for approximately 600 admissions annually of critically ill infants who require medical and surgical subspecialty care (Children’s Hospital Los Angeles, n.d.). The unit is designed with private and semi-private rooms to encourage families to stay with their infants and engage in caregiving activities. The NICU provides family-centered care with a broad range of interdisciplinary providers to support the infant and family during the hospitalization.

When an infant is medically stable enough to be transferred out of the NICU or Cardiothoracic Intensive Care Unit, the care team will refer the family to the Newborn Follow-up Program (NFP), a high-risk infant follow-up clinic. The rationale for such a clinic recognizes that newborns who experience critical illness or birth complications have a higher risk of brain injury and ongoing chronic medical problems that can lead to developmental delays and behavioral health risks. Initially, the program was established to align with the California Children’s Services (CCS) High-risk Infant Follow-up program (California Department of Health Care Services, n.d.). This program outlines eligibility criteria based on risks to brain development focusing on very low birth weight (less than 1500 g), premature birth (gestation length less than 32 weeks), and several neurologic, respiratory, infectious, cardiovascular and/or other medical issues. These conditions include chronic lung disease, conditions requiring extracorporeal membrane oxygenation (ECMO), congenital heart disease, seizures, stroke, meningitis, and others. The goal of the CCS program is to screen for developmental and behavioral needs and provide linkage to services. In the CHLA NICU, the referral is sent to the NFP Clinical Nurse Care Coordinator to explain the program to the family and schedule an appointment at the clinic.

The infant mental health and parent navigator services were funded by a grant from a philanthropic foundation and by billing to patient insurance.

### 2.3. Participants and Eligibility

All patients admitted to the NICU were eligible for Early Connections Program infant mental health services with the embedded psychologist, and all were eligible for Parent Navigator services (see details of interventions below). All patients receiving medical care in the NFP were eligible to meet with the NFP psychologist.

The unit of analysis was the individual infant. Each infant was represented by a separate record in the quality improvement database; twins and other multiples were therefore counted as separate patients. Because screening and intervention activities involved both the infant and the parent or caregiver, participating units are described as families.

### 2.4. Program Components and Intervention Procedures

#### 2.4.1. NICU Embedded Psychologist

The program has provided infant mental health support for NICU families since 2015, and since 2020, a full-time embedded psychologist has provided this service in the NICU. When infant mental health services began in the NICU, they were referral-based. Therapists working in the outpatient early childhood mental health program went to the NICU to provide services when requested by a NICU social worker. Qualitative interviews with stakeholders on the unit (neonatologists, nurse managers, and social workers) indicated that the service was seen as valuable to families. However, interviewees highlighted the wish for a universal approach in which every family could benefit and an embedded provider who could respond to family needs more readily and have more seamless communication with the medical team (Driver et al., 2021). Based on data from this qualitative study as well as the experience of the infant mental health team, the program changed its model by hiring a full-time psychologist to be embedded in the NICU. Co-location of infant mental health services within medical services has been recommended as a method to improve access to care (Hickey et al., 2024). In addition, changes were made to embrace a prevention approach in which all families are approached by the psychologist and offered services, rather than operating based on referral. This universal approach aims to enhance health equity by reducing barriers to access and avoiding potential bias that can occur when services are only offered to families who are identified by another provider and referred.

The NICU psychologist’s role has evolved into one that is deeply embedded in the daily rhythms of the unit, integrating a relationship-based, trauma-informed, and culturally sensitive approach to clinical interventions and interdisciplinary team processes and communications. The NICU psychologist offers mental health services to every family during hospitalization, with the goal of providing universal access to screening, early identification, and appropriate intervention tailored to the family’s unique needs. This proactive approach positions the psychology team’s services as a standard component of medical treatment, which aims to reduce stigma and shame associated with mental health and increase access to services that support infant development and nurture the caregiver-child relationship.

The model used by the Early Connections Program across settings, including in the NICU, has at its core an infant mental health framework, grounded in a relationship-based, trauma-informed, and culturally sensitive perspective. A central tenet of the approach is a focus on the parent–infant relationship as the primary target of intervention. The evidence-based practice guiding the intervention is Child–Parent Psychotherapy (CPP; Lieberman & Van Horn, 2008; Lieberman et al., 2015), a dyadic model designed for infants and young children who have experienced trauma or attachment disruptions. Our team has previously described applications of CPP to the NICU setting (Lakatos et al., 2019), including adaptations of the assessment and engagement and core intervention phases of treatment to fit the unique needs of families in the NICU. Services are provided in varying dosages depending on the clinical need, ranging from brief (one to two sessions) to interventions that continue throughout a lengthy hospital stay. The NICU psychologist completed the full clinical training and is rostered in CPP. For some families she completes the full CPP model and for others a briefer CPP-informed approach is utilized (as described in Lakatos et al., 2019). Regular individual and group reflective supervision by a mentor who is a CPP trainer is provided to the NICU psychologist.

Examples of CPP-informed interventions include, but are not limited to, co-creating empathic therapeutic alliances with caregivers by being present and bearing witness to their experience; developing a narrative to help the family make sense of their hospital journey; providing developmental guidance on reading and attuning to their baby’s cues (e.g., overstimulated vs engaged/ready to play, tiredness, discomfort due to environmental factors or physical symptoms/pain); and addressing the impact of trauma on the caregiver, the child, and their relationship. Further, dyadic interventions extend to include the child and family’s relationships with their medical providers; occupational, physical, and feeding therapists; lactation consultants; and psychosocial teams (e.g., Child Life specialists, chaplains, music therapists, and social workers) as every provider can foster parent–infant attachment through their work with families. The NICU psychologist also strives to create opportunities to engage in reflective conversations with interdisciplinary team members to support development and integration of an infant mental health perspective across disciplines.

#### 2.4.2. NICU Parent Navigator

The Parent Navigator model was originally developed in the outpatient setting (Mirzaian et al., 2024) in response to the difficulty families were experiencing obtaining early intervention for children with developmental delays or services for children with developmental disabilities. This model integrated parents with lived experience of having a child with a developmental disability in a pediatric primary care setting, where they were able to receive warm hand-offs from pediatricians and help families with all aspects of the process of connection to early intervention, including addressing hesitation and stigma by drawing on their lived experience, assisting with basic needs such as food, housing, and transportation that were potentially higher priorities for families, and assisting with practical challenges such as long applications to early intervention that require access to technology not available to all families (Mirzaian et al., 2024). This outpatient program achieved connection rates to early intervention of 71%, much higher than rates of 26–30% described in the literature in similar patient populations and settings (McManus et al., 2020; Jimenez et al., 2014).

Similar barriers to early intervention connection were witnessed in the program’s high-risk infant follow-up clinic (HRIF), referred to as the NFP, where at initial visit, despite 94.5% of patients meeting criteria for qualification for early intervention in this state based on a combination of biomedical risk factors, only 40% of patients had made a connection to early intervention (i.e., had made successful contact and were in the process of evaluation) (Mirzaian et al., 2022). Parents expressed in interviews regarding transition from the NICU to home that they wished there had been an “advocate” to help them with the process of early intervention, including having someone help them in the NICU before going home (Lakshmanan et al., 2022). Parents of infants discharged from the NICU have also commented about the importance of peer support and being able to talk to someone who also had a premature baby (Lakshmanan et al., 2019).

Given the successful outpatient model and the documented needs of NICU parents, the Parent Navigator model was implemented in the NICU at the end of 2025. The team posted a full-time paid employment position for a Parent Navigator, seeking an individual with lived experience as having a child who required hospitalization as an infant and also had familiarity with systems of care for patients with developmental delay. We were successful in recruiting a qualified individual, and she was trained over a 2 month period in line with California Department of Health Care Access and Information Community Health Worker (CHW) core competencies, the CHW, Promotores, and Representatives (CHW/P/R) Best Practices Framework (California Department of Health Care Access and Information, n.d.) Topics included motivational interviewing, social drivers of health, child development and developmental screening, positive parenting, the early intervention system, and support for basic needs including food, housing, and transportation. Clinical supervision with ongoing training and troubleshooting included weekly meetings with a supervising pediatrician as well as an infant mental health psychologist and communication with social work as needed.

This position was developed to work closely with the NICU team, including the psychologist, social workers, nurses, rehabilitation staff, and medical providers to play the unique role of connecting with parents by drawing on lived experience. The parent navigator’s primary focus is to provide education regarding programs that support well-being and development after discharge, specifically early intervention as well as the NFP. As both early intervention and the NFP focus on infant and toddler development, it can be confusing to parents and difficult to understand the need for both programs and logistical components of successfully connecting to each one. The Parent Navigator model in the NICU embeds a parent with lived experience of having an infant hospitalized for critical illness, who is trained in systems navigation and family support. The parent navigator develops a relationship with the family during the NICU hospitalization, typically close to discharge, assists with applications to early intervention prior to hospital discharge, and follows up with families after discharge via weekly or bi-weekly phone calls to ensure connection to early intervention is successful, NFP appointments are scheduled, and additional needs that arise after discharge are addressed, including social drivers of health and parental well-being through peer support. The Parent Navigator typically follows families for 3–6 months after NICU discharge.

### 2.5. Newborn Follow-Up Program: A High-Risk Infant Follow-Up Clinic

The interdisciplinary NFP team consists of a nurse care coordinator, pediatrician or developmental behavioral pediatrician, physical and occupational therapists, social worker, community health worker, and infant mental health psychologist. Expanding on the CCS high-risk infant follow-up’s requirement for three developmental visits over the first 3 years, infants in this hospital’s NFP are seen soon after they are discharged from the hospital to introduce them to the team and identify any immediate needs. They are then seen at the subsequent visits of approximately 6 months, 12 months, and 24 months of age corrected for prematurity. An additional visit is possible between 2 and 3 years of age if there are needs for further follow-up or to ensure linkage to transition services from the early intervention system into the schools. While the focus of the visits is the medical history, neurological examination, and developmental assessment, the family’s health-related social needs, barriers to services, and parent–infant dyadic functioning are also assessed. The clinic visit includes evidence-based developmental assessment using tools appropriate for the child’s age. The recommendations include initiation or modification of early intervention services, referrals to medical providers, linkage to social services, engagement in dyadic or parental support groups, and transition to school-based resources. The NFP specialists see about 400 patients across a seven-county area each year.

#### Newborn Follow-Up Program Embedded Psychologist

In this universal model of care, the infant mental health psychologist embedded in the NFP is an integral part of the interdisciplinary team and offers all families behavioral health screening and consultation. The psychologist’s role is to briefly assess infant mental health, including dyadic relational functioning; determine if there are parent, infant, or relational needs or risk factors; and provide linkage to the appropriate level of intervention. The levels of intervention (i.e., universal, targeted, and clinical) were developed based on the Pediatric Psychosocial Preventative Health Model (Kazak, 2006; Kazak et al., 2024) and stepped-care approaches (De Young et al., 2021). In late 2025, the NFP psychologist began piloting infant mental health screening using portions of the Survey of Well-being in Young Children (SWYC; Perrin et al., 2016) (see Measures for details) to improve identification of infants and young children at risk of or with existing behavioral health needs.

The psychologist uses the behavioral health screener, clinical interview with the parent, observations of the infant and dyad, and consultation with the interdisciplinary team to inform interventions and recommendations. The psychologist meets with the family to conduct a clinical interview using open ended questions to learn about the family’s experience in the intensive care unit, how they have adjusted to the transition home, and whether there are any lingering symptoms of anxiety, depression, or posttraumatic stress now that they are home. If parents report mental health symptoms, the psychologist asks about any current adult mental health services received and offers linkage to perinatal mental health services through the Early Connections Program or community resources. In addition, a parent education handout developed by the Early Connections Program provides information about common adult mental health symptoms when caring for a baby with a medical condition and community resources including support groups and how to find a therapist. The psychologist asks about and observes the infant’s temperament, signs of medical traumatic stress, and the parent’s ability to read the infant’s cues, as well as behavioral and social–emotional concerns.

The psychologist provides the universal-level intervention to all families who attend their NFP appointment and agree to meet with the psychologist. This includes the clinical screening and assessment described above, which is considered to be a clinical intervention in itself because it is conducted by an infant mental health specialist who uses the interview and discussion of the screening tool as a way to provide trauma-informed empathic listening and developmental guidance. Additional universal interventions are specific developmental and parenting guidance and parent education materials. For families with mild-to-moderate areas of concern identified during the consultation and/or on the behavioral health screener, a targeted intervention is offered, which includes either a parent support and educational group, brief home visiting services, or referral to a community-based support group. For families with more significant needs, clinical intervention is offered through the Early Connections Program’s outpatient behavioral health service, including dyadic infant mental health therapy and perinatal mental health services for parents of infants. Currently, decisions about what level of intervention to offer are based on the psychologist’s clinical judgement in collaboration with the family and input provided by other interdisciplinary team members. Over time, we plan to more systematically measure the decision-making process for selecting a universal, targeted, or clinical intervention.

## 3. Measures

### 3.1. Survey of Wellbeing of Young Children

The Survey of Wellbeing of Young Children (SWYC; Perrin et al., 2016) is a parent-completed screening questionnaire designed for children ages 1 to 66 months. The full measure includes developmental milestones, social–emotional functioning, symptoms of autism spectrum disorder, and questions about family risk factors. For this project, the social–emotional section of the SWYC was used, which includes either the Baby Pediatric Symptom Checklist (BPSC; 12 items; administered to children aged 1 to 18 months) or the Preschool Pediatric Symptom Checklist (PPSC; 18 items; administered to children aged 19 to 36 months). Chronological age was used when selecting the measure, because gestational age was not available in the electronic medical record in a format that allowed for selection of measures based on gestational age. This means that some children in the 19- to 22-month age range may have been administered the PPSC when the BPSC would have been the appropriate measure. The BPSC has three subscales, and the measure is in the at-risk range if any of the subscales has a score of 3 or greater. The PPSC has only one score, and a score of 9 or higher is in the at-risk range. The PPSC was validated on a sample of about 800 children, showing comparability with other parent-report measures of social–emotional functioning in young children and indicating no differences in screening accuracy based on parent education or child race, ethnicity, sex, or age (Sheldrick et al., 2012). The BPSC has been found to have a stable factor structure, similar responses across child characteristics (e.g., race, ethnicity, sex, and urban vs rural setting), and comparability with other parent-report measures (Sheldrick et al., 2013; Zablotsky et al., 2023).

#### 3.1.1. Data Collection

##### SWYC BPSC and PPSC

For the program described here, the English and Spanish versions of the BPSC and PPSC were inputted into the REDCap system (Nashville, TN, USA; Harris et al., 2009), which enabled electronic completion of the measure by the parent. Using the REDCap system, the measure was sent to the family prior to the scheduled appointment through their email and/or phone by text. When the parent completed the measure, it was automatically scored and uploaded into the child’s electronic medical record. In addition, the REDCap system automatically sent an email to the infant mental health psychologist assigned to the clinic, informing them that the measure had been completed and providing the scores. If the parent did not complete the screener in advance, the clinician had the option to administer it in person during the appointment by using a tablet, sending a link to the parent for them to complete on their phone, providing a paper version of the measure, or administering the measure through interview. For families who did not speak or read English or Spanish, interpreter support was available for conducting a clinical interview and the screening tool was not used.

### 3.2. Screening and Intervention Tracking System

For quality improvement purposes, the program developed a tracking system to collect detailed information about the screening and interventions provided by the infant mental health psychologists in the NICU and in the NFP. The tracking system was used in the NICU for a 3-month period (1/1/26 to 31/1/26) and in the NFP for a 4-month period (1/12/25 to 31/3/26). These time frames differed due to implementation of the tracking system in the NFP first, a month prior to implementation of the tracking system in the NICU. The tracking system was deployed in a staged manner to ensure it was functional in one clinic, prior to introduction into a second area of the program. The program coordinator entered data into the tracking system for all children admitted to the NICU and all children scheduled for an appointment in the NFP (including name, medical record number, date of birth, and for NFP, the date of appointment and the date the screener was sent to the family). In the NFP, after each clinic, the psychologist documented the following variables for each patient: what type of screening was completed (interview and observation and/or the SWYC); whether the screener was completed in advance or at the appointment; if completed at the appointment, the method of completion (iPad, paper, electronic link sent to parent); and the reason if no SWYC was completed (e.g., did not attend appointment; psychologist absent from clinic; parent declined to complete measure; psychologist decided not to administer measure). For both NICU and NFP, the psychologist documented the following variables for each patient: what interventions were provided to the family (from a drop-down list of options) and the reason if no intervention was provided. Data was checked by the Early Connections Director to ensure that an entry was made by the psychologist for each patient entered in the tracking system.

#### 3.2.1. Data Analysis

All analyses were descriptive. Categorical variables were summarized using frequencies and percentages. Unless otherwise specified, percentages were calculated using the number of families with available data for the relevant measure as the denominator. Screening results were summarized separately for the age-appropriate Baby Pediatric Symptom Checklist and Preschool Pediatric Symptom Checklist. For items in which more than one response could be selected, categories were treated as non-mutually exclusive; therefore, percentages could total more than 100%. Missing or incomplete responses were not imputed, and analyses were based on available data. The denominator used for each analysis is reported in the corresponding tables. No inferential statistical analyses were performed. Data were managed and analyzed using SAS v9.4 (Cary, NC, USA).

#### 3.2.2. Ethical Considerations

The project was submitted to the CHLA Institutional Review Board and was determined to be quality improvement, not research.

## 4. Results

Prior to implementation of the quality improvement project, the NICU psychologist focused on families referred by a social worker for infant mental health services. For the new pilot program, the NICU psychologist attempted to reach all families admitted to the NICU to offer services. There were a total of 114 patients in the NICU during the three-month tracking period (1/1/26 to 31/3/26). Of these, 63 (55.3%) families were seen by the infant mental health psychologist. For the quality improvement evaluation, reasons that families were not seen were examined and included: discharged before attempted contact (*n* = 31, 27.2%), attempted but unable to make contact (*n* = 12, 10.5%), or parent declined to meet with the psychologist (*n* = 8, 7.0%). All families who could be contacted were provided with information about the psychologist’s role and their availability for support during the hospitalization. Of those who met the psychologist, 56 (88.9%) had one infant mental health family session, 41 (65.1%) had additional follow-up infant mental health sessions during the hospital admission, and 7 (11.1%) declined further services after hearing about the program. To further improve the reach of the program, the psychologist began making phone calls to families who were not at bedside and requesting assistance from the unit social workers to facilitate linkage. Future analyses will examine more closely the specific reasons for families not being at bedside and the effectiveness of alternative approaches to reach them.

Prior to the quality improvement effort, no parent navigator services were available in the NICU. During the pilot, a total of 66 families (57.9%) in the NICU were supported by the parent navigator during the three-month tracking period (1/1/26 to 31/3/26). All families were eligible for parent navigator services, but some were not seen because they could not be reached by the parent navigator (e.g., not at bedside and not responding to phone calls), or they declined to meet with her. Sixty families (90.9% of those who met with the parent navigator) submitted an application to early intervention with the assistance of the parent navigator. The outcome of the application was not available yet at the time of this analysis. Future analyses will document the number and proportion of children completing the early intervention evaluation, found eligible for services, and successfully beginning services. Depending on the findings from those analyses, we will further refine the approach to address barriers to linkage.

Prior to the launch of the quality improvement project, the psychologist in the NFP focused on families having their first visit to the clinic and families flagged by another member of the clinic team as needing psychological support. In addition, no formal screening measure was used. For the new pilot program, the screening measure was sent to every family scheduled for an appointment in the week prior to the scheduled clinic. In addition, the psychologist attempted to meet with every family scheduled for a clinic visit. There were 168 families who had been discharged from either the NICU or Cardiothoracic Intensive Care Unit and had appointments in the NFP during the four-month pilot period (1/12/2025 to 31/3/2026), and 114 (67.9%) were screened by the embedded psychologist using clinical interview, observation, and/or the SWYC. As for the SWYC screening, 85 (74.6%) of the 114 patients who met with the psychologist were screened using the tool, meaning that approximately half (50.6%) of all families who had scheduled appointments in NFP during this period were screened with the SWYC. Fifty-four (32.1%) families were not seen by the psychologist due to one of the following reasons: family no-show (*n* = 27, 50.0%), clinician was unavailable on the clinic date (*n* = 14, 25.9%), or insufficient time to see all patients during clinic (*n* = 12, 22.2%). For one patient (1.9%) the reason was not documented. In order to improve reach, we plan to assign other psychologists from our team to cover the NFP clinic during scheduled vacations or sick days of the NFP psychologist and to follow up by phone with families who did not show up for the clinic or who could not be seen on the day of the clinic due to higher volume of sessions and needs on the clinic day.

Of those 85 (50.6%) families who completed the screening measure in the NFP, 13 (23.6%) infants scored over the at-risk cutoff for the BPSC and 12 (40.0%) toddlers scored over the at-risk cutoff for the PPSC (Table 1). Table 2 depicts the BPSC distribution by subscale.

Because chronological age was used to determine which screening measure to administer (due to limitations in access to gestational age in electronic form in the medical record), we did a post hoc review of specific charts to determine which children were administered the PPSC when they should have been administered the BPSC based on corrected age. We identified three children who were administered the incorrect measure. All three scored below the at-risk cutoff for the PPSC.

Of the 114 families who were screened (through SWYC and/or clinical interview), 23 (20.2%) were also referred for targeted intervention. Those interventions included the parent group offered through the program (9 families), home visiting offered through the program (4 families), additional follow-up sessions with the NFP infant mental health psychologist (1 family), and referral to a community resource such as through early intervention or a community support group (9 families). Finally, 4 (3.5%) families were linked with a clinical intervention provided by the program’s outpatient dyadic therapists. The decision about what intervention to offer was made by the psychologist considering all information gathered, the family’s preferences, and whether they were already involved with behavioral health services or other supports in the community. In this sample, there was no overlap in families referred for targeted and clinical interventions.

## 5. Discussion

Across inpatient and outpatient medical settings, there are opportunities to support infants and families who have experienced a NICU admission and the accompanying stressful, potentially traumatic, events related to hospitalization and subsequent outpatient medical care. As part of the Early Connections Program, tiered infant mental health interventions have been developed that align with the Pediatric Psychosocial Preventative Health Model (PPHM) along the levels of universal, targeted, or clinical intervention (Kazak, 2006). Developed infant mental health interventions tailored to the infant and family’s level of need, ranging from one-time psychoeducational materials to long-term CPP dyadic clinical intervention, allow the Early Connections provider to flexibly provide the interventions that meet a family’s current level of need. By implementing a universal model that is available to all families, an embedded NICU psychologist can introduce their role to the family as part of the standard of care while the infant is admitted to the hospital; this approach aims to decrease stigma and normalize the understandable stress experienced by families. Very few families refused services from the embedded NICU psychologist. Based on data entered by clinicians into the tracking system, the main barriers to engaging families in services were discharge prior to being able to meet with the psychologist due to a short length of stay and challenges making contact with the family when the infant was hospitalized. Although data was not collected on the specific contributing factors to these barriers, anecdotally, some families have difficulty coming to the NICU during the week due to work commitments, the necessity of caring for other children, and living far from the hospital. Efforts to leverage technology such as telephone and telehealth sessions may work to reduce these barriers. Coordination with other NICU providers, such as social workers or nurses, may also be helpful in identifying a family’s specific schedule regarding hospital visits and potential windows of time families will be onsite to attempt to engage and support more families.

Over half of families admitted to the NICU received services from the embedded psychologist, and 65% of those families received subsequent intervention sessions. The current results support previous findings regarding the importance of flexibility in having a dose-dependent approach when applying CPP in a NICU setting (Driver et al., 2021). Some families engage in one session with an embedded NICU psychologist, while other families receive more intensive services as their infant’s trajectory may change across the course of hospitalization.

The Parent Navigator model was implemented in the NICU at the end of 2025. To date, our results indicate that the parent navigator has been a positive resource and support to families navigating early intervention for their infant following discharge from the NICU. It is encouraging that the overwhelming majority of families who met with the parent navigator have successfully completed an application for early intervention services. Given the relatively recent implementation of the parent navigator in the Early Connections Program and the length of time it takes for an assessment to be completed after an application is submitted to early intervention services, the percentage of those infants who qualify for services is currently unknown but will be reported when that data is available. However, given the high rate of completed applications to date, a parent navigator in the NICU who provides support after the family is discharged home may help families connect with early intervention services, as has been demonstrated by parent navigators in outpatient primary care and similar settings (McManus et al., 2020; Jimenez et al., 2014). Given the increased risk for neurodevelopmental challenges for infants who were admitted to the NICU and the benefit of early intervention, a parent navigator is an additional strategy to support the well-being of infants and families in accessing needed developmental services as they transition home from the hospital.

After transitioning to a universal model of infant mental health services, more than half of families met with the embedded psychologist in the NFP for clinical interview, observation and/or completion of the SWYC. However, there were families who were not seen by the psychologist due to not attending their NFP appointment, the psychologist being unavailable during a clinic, or time constraints during the NFP appointment. The NFP administrative staff and psychologist are exploring telehealth options for the psychologist to connect with families following their NFP appointments when time constraints during the NFP clinic are a barrier or if the psychologist is unavailable on a specific clinic date. Additionally, the Early Connections program instituted a coverage plan in which infant mental health psychologists are available to provide coverage for each other when one provider is unavailable. To optimize the family’s and providers’ time in the NFP clinic, the SWYC is also sent to families ahead of appointments to reduce the time burden of a family completing the SWYC during their appointment. For families who did not attend their appointment, the psychologist will be available to see the family if they attend a rescheduled appointment in NFP. Given that approximately half of the families seen in NFP completed the SWYC, results cannot be generalized to describe the entire population of infants and families supported in high-risk follow-up clinics. However, SWYC results in the NFP reflected that almost a quarter of the infants and 40% of the toddlers who were screened scored in the range suggesting risk for social-emotional concerns. While the results have been mixed in the literature about behavioral difficulties in children who are born pre-term (Shaw et al., 2023), the preliminary results from this project support the need for ongoing, repeated social–emotional screening for infants and young children who have medical complexity that required admission to intensive care.

The current quality improvement project focused on the implementation of support across inpatient and outpatient settings for families whose child was hospitalized in an infant critical care unit. This project reports on family engagement with and utilization of a universal model of care provided by an embedded psychologist in the NICU, the incorporation of a parent navigator to assist families in completing the application process for early intervention services, and the implementation of social–emotional screening in the NFP. Future directions include identifying the system-related and family-related barriers to connecting with families across both inpatient and outpatient settings to address barriers and to maximize the impact of this screening and intervention program. The parent navigator has successfully engaged families to complete and submit applications for early intervention; however, that is the first step in the eligibility process. Additional information is needed to determine if the infants were deemed eligible and obtained services. Based on the level of emotional and behavioral risk reflected in the families and young children screened in the NFP, future research can contribute to understanding if there are differences in social–emotional risk between the infant and toddler age ranges in NFP and the potential impact of development on differences in level of risk. Specific attention will be given to developing tailored interventions to address areas of concern in that population of families of infants and toddlers with special healthcare needs.

### Limitations

This quality improvement project was implemented in a single academic medical center, and findings may not be generalizable to hospitals with different patient populations, resources, staffing, or clinical services. The programs also relied substantially on philanthropic support, despite some reimbursement through insurance, which may limit feasibility in other settings. Because the findings represent a brief pilot period, longer-term evaluation is needed as program reach increases. The absence of pre-implementation comparison data and the descriptive nature of the analysis also preclude conclusions about program effectiveness.

The SWYC is a family-completed self-report measure and may be subject to social desirability, underreporting, or overreporting, although screening was supplemented or replaced, when appropriate, by interviews and clinical observations conducted by a psychologist trained in infant mental health. Some families encountered barriers to completing the SWYC or omitted items, resulting in missing data. Incomplete program reach and screening completion may have introduced selection bias because participating families may differ from families who were not reached or did not complete screening, potentially limiting generalizability and underestimating developmental or psychosocial needs.

Chronological rather than corrected age was used to select the SWYC form because gestational age was available only within narrative clinical notes and could not be incorporated into the automated electronic administration process. This approach may overidentify or misclassify social–emotional risk among children born preterm. Although a limited post hoc chart review did not identify false-positive results among children near the PPSC age cutoff, this is an important limitation to consider when interpreting those measures.

Finally, the project was designed to assess overall implementation rather than follow families longitudinally through each service pathway. Available data did not consistently capture pathway-specific eligibility, contact attempts, session completion, SWYC screening, or subsequent intervention tiers, limiting pathway comparison and preventing construction of a complete pathway-level flow diagram. There is a lack of pre-implementation data as a comparator, and the data reflected from this project improvement project is descriptive in nature. Reliance on clinician-entered data may also have resulted in incomplete documentation, inconsistent reporting, or misclassification of contacts, screening completion, and services delivered.

## 6. Conclusions

In summary, the Early Connections Program provides multiple opportunities to support infants and their families, bridging from inpatient to outpatient healthcare settings. As the needs of families and infants may change over time, it is important to have multiple touch points where needs can be assessed flexibly, via interview and a standardized screening tool, and the appropriate level of tiered intervention can be provided based on level of need. This model demonstrates the potential for integrating infant mental health across inpatient and outpatient settings. Preliminary quality improvement data provides information on program reach, family engagement, screening processes to aid in determining the level of intervention provided, and the support of a parent partner in navigating systems after discharge from the hospital.

## Figures and Tables

**Table 1:** Completed SWYC outcomes (*n* = 85).

Measure	Within Normal Range	Over Cutoff
Baby Pediatric Symptom Checklist (ages 1–18 months) (*n* = 55)	42 (76.3)	13 (23.6)
Preschool Pediatric Symptom Checklist (ages 19–36 months) (*n* = 30)	18 (60.0)	12 (40.0)

**Table 2:** Distribution of scores in Baby Pediatric Symptom Checklist by subscale (ages 1–18 months) (*n* = 55) *.

Subscale	N	Percent (%)
BPSC Q1–4 (Novelty/Irritability) (*n* = 54)		
0	27	50.0
1	11	20.4
2	7	13.0
3+ (at-risk range)	9	16.7
BPSC Q5–8 (Regulation/Inflexibility) (*n* = 55)		
0	25	45.5
1	11	20.0
2	10	18.2
3+ (at-risk range)	9	16.4
BPSC Q9–12 (Difficulty with Routines) (*n* = 55)		
0	27	49.1
1	12	21.9
2	6	10.9
3+ (at-risk range)	10	18.2

* Percentages were calculated using the number of available responses for each subscale. One response was missing for BPSC Q1–4 (*n* = 54); complete data were available for BPSC Q5–8 and Q9–12 (*n* = 55). Percentages may not total 100% because of rounding.

## Data Availability

The data were collected and analyzed as part of an institutional quality improvement initiative. The authorization for this project did not include external distribution of the underlying record-level data, which are also subject to institutional privacy and data-governance restrictions.
